# A Preliminary Evaluation of Middle Ear Resonance Frequency via Wideband Tympanometry in Patients with Non-Alcoholic Fatty Liver Disease

**DOI:** 10.3390/jcm15145335

**Published:** 2026-07-08

**Authors:** Mustafa Taştan, Alihan Oral, Tuğba Yemiş

**Affiliations:** 1 Department of Otorhinolaryngology-Head and Neck Surgery, Faculty of Medicine, Istinye University, Istanbul 34010, Türkiye; mustafa.tastan@istinye.edu.tr; 2 Department of Internal Medicine, Faculty of Medicine, Biruni University, Istanbul 34010, Türkiye; aoral@biruni.edu.tr; 3 Department of Otorhinolaryngology-Head and Neck Surgery, Faculty of Medicine, Recep Tayyip Erdogan University, Rize 53100, Türkiye

**Keywords:** wideband tympanometry, non-alcoholic fatty liver disease, resonance frequency, systemic inflammation, middle ear absorbance

## Abstract

**Background and Objective:** Non-alcoholic fatty liver disease (NAFLD) induces systemic low-grade inflammation and lipotoxicity. While its microvascular effects on the inner ear are recognized, its impact on the middle ear remains unexplored. This study aimed to investigate subclinical biomechanical alterations in the middle ear of normal-hearing NAFLD patients using Wideband Tympanometry (WBT). **Materials and Methods:** This prospective observational study included 28 patients with ultrasonographically confirmed NAFLD and 30 healthy, sex-matched controls. All included participants had normal pure-tone hearing thresholds and Type A tympanograms. WBT was performed to measure Resonance Frequency (RF) and frequency-specific absorbance (226–8000 Hz). A subgroup analysis compared early-stage (Grade 1) and advanced-stage (Grades 2–3) hepatosteatosis. **Results:** The mean RF was significantly lower in the NAFLD group compared to the controls for both the right (781.6 ± 218.7 vs. 1010.6 ± 529.0 Hz; *p* = 0.035) and left ears (740.3 ± 212.7 vs. 1221.6 ± 754.2 Hz; *p* = 0.002). Furthermore, wideband absorbance was significantly elevated at lower frequencies (250 Hz and 500 Hz) in the study group (*p* < 0.001 for both ears). Middle ear resting pressures were similar between the groups. Subgroup analysis revealed no significant differences in WBT parameters between Grade 1 and advanced stages (*p* > 0.05). **Conclusions:** Normal-hearing NAFLD patients exhibit a mass-dominated biomechanical shift in the middle ear, characterized by a decreased RF and increased low-frequency absorbance. These subclinical alterations, observable even in early-stage hepatosteatosis, likely reflect mucosal micro-edema driven by systemic inflammation. WBT serves as a sensitive, non-invasive tool for detecting early metabolic-related otologic changes.

## 1. Introduction

Nonalcoholic fatty liver disease (NAFLD) is increasingly recognized not only as an isolated liver disease, but as a complex metabolic disorder with systemic implications [1]. The pathogenesis of NAFLD is closely linked to lipotoxicity, insulin resistance, and chronic low-grade inflammation [2,3]. Emerging evidence suggests that the excessive accumulation of hepatic lipids triggers the activation of potent pro-inflammatory pathways, most notably the sphingomyelinase–ceramide axis [4]. This activation releases a cascade of inflammatory mediators into the bloodstream, which can eventually compromise the microvascular and mucosal integrity of distant organs.

In the field of otolaryngology and audiology, the detrimental effects of metabolic syndromes, obesity, and diabetes on the auditory system have been widely investigated, though research has mostly focused on the inner ear [5,6]. Furthermore, a broader paradigm shift in hearing research suggests that audiologic evaluations should not be limited solely to local ear-related issues; systemically, vascular risk factors and serum electrolyte levels also play critical roles in the pathogenesis of hearing loss [7,8]. Numerous studies link metabolic disturbances to sensorineural hearing loss, generally pointing to cochlear microvascular damage and subsequent hair cell apoptosis [9]. However, the impact of systemic low-grade inflammation on the middle ear and Eustachian tube mucosa has received very little attention. It is highly possible that the circulating inflammatory mediators present in NAFLD could lead to subclinical mucosal edema and microvascular congestion in the tympanic cavity, altering its biomechanics.

Detecting these minor, subclinical changes is difficult with standard hearing tests. Conventional pure-tone audiometry (conducted within the frequency range of 125 to 8000 Hz) and classical 226 Hz tympanometry often lack the sensitivity needed to catch early biomechanical shifts before they manifest as overt conductive hearing loss. Wideband Tympanometry (WBT), on the other hand, offers a more advanced and sensitive approach [10]. Tympanometry remains a fundamental, objective, and non-invasive tool in otorhinolaryngological and audiological diagnostics, crucial for evaluating middle ear pathologies. However, while conventional tympanometry is limited to a single 226 Hz probe tone providing static compliance data, wideband tympanometry (WBT) offers a comprehensive analysis across a broad frequency spectrum. Furthermore, acoustic reflex measurements performed during immittance testing provide vital clinical insights into the stapedius muscle contraction, middle ear compliance dynamics, and neural pathway integrity. By measuring the acoustic immittance of the middle ear across a wide range of frequencies (typically 226 to 8000 Hz), WBT can track dynamic parameters like wideband absorbance and Resonance Frequency (RF). This allows clinicians to detect very subtle changes in the mass and stiffness of the middle ear system.

In this study, we hypothesized that the systemic lipotoxicity and inflammation associated with NAFLD might cause subclinical micro-edema in the middle ear mucosa, leading to early biomechanical alterations even in the absence of subjective hearing complaints. We aimed to use WBT to investigate subclinical middle ear biomechanical changes in normal-hearing patients with ultrasonographically confirmed hepatosteatosis, and to compare the findings with a healthy control group.

## 2. Materials and Methods

### 2.1. Study Design and Participants

This prospective observational clinical study was conducted at the Department of Otorhinolaryngology. The study protocol was approved by the Biruni University Clinical Research Ethics Committee (Approval No: 2026-07; Date: 1 July 2026), and written informed consent was obtained from all participants in accordance with the Declaration of Helsinki.

The study cohort consisted of 58 adults. Participants were consecutively recruited from the internal medicine outpatient clinic. Patients who underwent abdominal ultrasonography and were diagnosed with hepatic steatosis were referred to the otorhinolaryngology department for further evaluation. Following a detailed medical history and criteria screening, eligible patients were assigned to either the NAFLD group (*n* = 28) or the healthy control group (*n* = 30). For the healthy control group, the absence of hepatic steatosis was objectively confirmed via abdominal ultrasonography, alongside normal liver function tests and no history of chronic metabolic or systemic diseases.

### 2.2. Inclusion and Exclusion Criteria

The study included participants aged between 18 and 45 years. All participants were required to have normal otoscopic examination findings, an intact tympanic membrane, a Type A tympanogram on standard 226 Hz tympanometry, and normal pure-tone hearing thresholds (defined as ≤20 dB HL across the 250–8000 Hz frequency range). Patients were excluded from the study if they had a history of otologic surgery, chronic otitis media, tympanic membrane perforation, otosclerosis, active upper respiratory tract infections, or any degree of conductive or sensorineural hearing loss (air-bone gap > 10 dB). Participants in the healthy control group were not taking any regular medications, including systemic anti-inflammatory drugs, ototoxic medications, or treatments for metabolic diseases, ensuring an unconfounded baseline for wideband tympanometry measurements. Furthermore, individuals with a history of active smoking, a history of regular alcohol consumption, allergic rhinitis, chronic sinusitis, or nasal polyposis were excluded. Finally, patients with coexisting systemic conditions, including diabetes mellitus, essential hypertension, autoimmune disorders, or chronic kidney disease, were also excluded from the study cohort.

### 2.3. Radiological and Biochemical Evaluations

In the study group, the diagnosis and grading of hepatosteatosis (Grades 1 to 3) were confirmed via abdominal ultrasonography (USG) performed by an experienced radiologist according to well-established clinical criteria [11]. Fasting venous blood samples were collected from all participants in both groups between 08:00 and 10:00 AM following an overnight fast of at least 8 h. The biochemical parameters analyzed using standard automated laboratory assays included aspartate aminotransferase (AST), alanine aminotransferase (ALT), gamma-glutamyl transferase (GGT), total cholesterol, high-density lipoprotein (HDL), low-density lipoprotein (LDL), triglycerides, and fasting glucose.

### 2.4. Audiological and Wideband Tympanometry (WBT) Measurements

Following the otorhinolaryngological examination, pure-tone audiometry was performed using a clinical audiometer (AC40; Interacoustics, Middelfart, Denmark) in a sound-attenuated booth. Air conduction thresholds were measured across the frequency range of 125 to 8000 Hz, while bone conduction thresholds were evaluated between 250 and 4000 Hz. Standard clinical masking procedures, including masked-bone conduction assessments, were systematically employed whenever a significant air–bone gap or interaural attenuation threshold was detected. Subsequent wideband acoustic immittance measurements were conducted using a wideband tympanometry system (Titan; Interacoustics, Middelfart, Denmark). Additionally, acoustic reflex testing (including both ipsilateral and contralateral pathways) was systematically conducted for each individual case in both the NAFLD and control groups, using the same system to ensure comprehensive middle ear and neural pathway evaluation. Wideband absorbance (ranging from 0 to 1) was measured across a frequency spectrum of 226 to 8000 Hz. The primary parameters extracted for statistical analysis included Resonance Frequency (RF) in Hertz (Hz), wideband absorbance values at specific frequencies (250, 500, 1000, 2000, 4000, and 8000 Hz), Peak Absorbance, Peak Absorbance Frequency, and middle ear pressure (daPa) at 226 Hz. Measurements were recorded separately for the right and left ears.

### 2.5. Statistical Analysis

Statistical analyses were performed using SPSS software, version 26.0 (IBM Corp., Armonk, NY, USA). The normality of the data distribution was evaluated using the Shapiro–Wilk test. Normally distributed continuous variables were expressed as mean ± standard deviation (SD), and categorical variables were reported as frequencies and percentages. The independent samples *t*-test was utilized to compare continuous variables, including age and wideband tympanometry parameters, between the study and control groups. The Chi-square test was employed to compare sex distribution. For the subgroup analysis within the NAFLD cohort (Grade 1 versus Grades 2–3), the independent samples *t*-test was applied. A two-sided *p*-value of <0.05 was considered statistically significant.

## 3. Results

### 3.1. Demographic and Clinical Characteristics of the Participants

The present study included a total of 58 participants. The study group consisted of 28 patients diagnosed with non-alcoholic fatty liver disease (NAFLD), while the control group comprised 30 healthy volunteers. The baseline demographic and clinical characteristics of the groups are summarized in Table 1.

There was no statistically significant difference between the study and control groups in terms of sex distribution (*p* = 0.746). A statistically significant difference was observed in the mean age of the participants, which was 36.8 ± 7.3 years in the study group and 30.7 ± 7.3 years in the control group (*p* = 0.002). Regarding the severity of hepatic fat accumulation in the study group, 53.6% of the patients presented with Grade 1 hepatosteatosis, followed by Grade 2 (32.1%) and Grade 3 (14.3%).

### 3.2. Wideband Tympanometry (WBT) Findings

**Figure 1:**
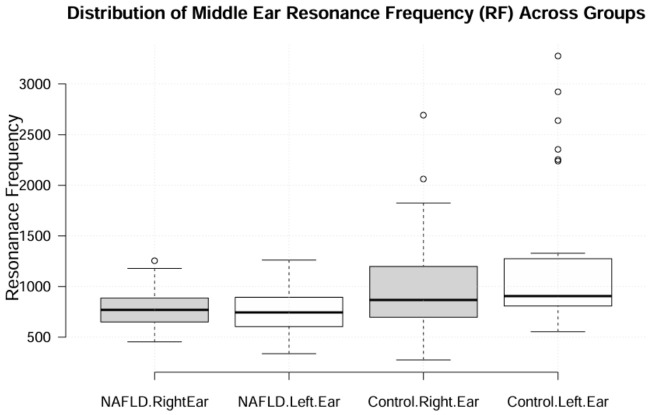
Boxplot comparison of middle ear Resonance Frequency (RF) between the NAFLD and healthy control groups for both the right and left ears. The central horizontal lines indicate the medians, the boxes represent the interquartile ranges (IQR), and the whiskers show the data variability.

Wideband acoustic immittance measurements demonstrated significant differences between the study and control groups. The WBT parameters for the right and left ears are detailed in Table 2 and Table 3, respectively. The mean Resonance Frequency (RF) values were significantly lower in the hepatosteatosis group compared to the healthy controls for both the right ear (*p* = 0.035) and the left ear (*p* = 0.002). The exact distribution, medians, and interquartile ranges of the resonance frequencies across the groups are visually demonstrated in Figure 1.

Furthermore, frequency-specific absorbance analyses revealed significant elevations at lower frequencies in the study group. Specifically, for 250 Hz, absorbance was significantly higher in both the right ear (*p* < 0.001) and left ear (*p* < 0.001). Similarly, for 500 Hz, the significant elevation was firmly maintained for both the right ear (*p* < 0.001) and left ear (*p* < 0.001). The frequency at which peak absorbance occurred was also significantly shifted toward lower frequencies in the NAFLD patients. There were no statistically significant differences between the groups regarding middle ear resting pressure at 226 Hz or high-frequency absorbance values.

To evaluate whether the baseline age difference between the study groups influenced the wideband acoustic immittance parameters, a secondary Analysis of Covariance (ANCOVA) was performed by introducing age as a covariate. The ANCOVA results demonstrated that the statistical significance of all primary parameters was firmly maintained. Notably, the differences in resonance frequencies remained highly significant even after adjusting for age (*p* = 0.01 for the right ear, *p* = 0.001 for the left ear; Figure 1), and low-frequency absorbances at 0.25, 0.5 and 1 kHz also remained highly significant (*p* < 0.05), confirming that the findings were independently associated with NAFLD status rather than age variance.

### 3.3. Subgroup Analysis Based on Hepatosteatosis Grading

To evaluate whether the severity of hepatic fat accumulation correlated with middle ear biomechanical alterations, a subgroup analysis was conducted within the study group. The NAFLD patients were categorized into two subgroups: Grade 1 hepatosteatosis (*n* = 15) and advanced (Grade 2 or 3) hepatosteatosis (*n* = 13). The comparison of key wideband acoustic immittance parameters is presented in Table 4. There were no statistically significant differences between the Grade 1 and Grade 2–3 subgroups in terms of Resonance Frequency or Peak Absorbance Frequency for either ear (*p* > 0.05). Furthermore, wideband absorbance values at 250 Hz and 500 Hz showed no significant differences between the subgroups.

## 4. Discussion

Our primary findings indicated that normal-hearing non-alcoholic fatty liver disease (NAFLD) patients exhibited a significant decrease in middle ear Resonance Frequency (RF) and a simultaneous increase in wideband absorbance at lower frequencies (250 Hz and 500 Hz) compared to healthy controls. Currently, there are very few studies in the literature investigating the relationship between NAFLD and the middle ear mechanics. The present study is one of the few to specifically examine this association. Although studies specifically investigating middle ear biomechanics via wideband tympanometry in NAFLD are exceptionally scarce, our findings align conceptually with earlier reports demonstrating the negative impacts of metabolic dysfunction and abdominal fat accumulation on the auditory system. The previous literature has indicated that chronic inflammation, insulin resistance, and systemic metabolic stress—which form the core pathophysiology of hepatic steatosis—can lead to subclinical neural and cochlear impairments, often manifested as elevated pure-tone thresholds or sensorineural hearing loss [12,13]. While those earlier reports primarily focused on the sensory and neural components of hearing, our study expands this paradigm by demonstrating that the metabolic burden of NAFLD also alters the peripheral conductive mechanics of the middle ear, as objectively shown by WBT. The simultaneous presence of subclinical hearing deficits reported in the past literature and the low-frequency acoustic absorbance elevations and reduced resonance frequencies observed in our cohort further substantiate the hypothesis that NAFLD-associated lipotoxicity and systemic inflammation exert a widespread impact on the entire auditory system, ranging from middle ear compliance to cochlear and neural function. These findings collectively suggest that the middle ear mucosa, like the cochlea, is vulnerable to the systemic inflammatory burden of metabolic diseases, positioning WBT as a promising non-invasive screening tool for early otologic manifestations of systemic metabolic disorders. In our study, we also conducted subgroup analysis which suggested that mass-dominated biomechanical shifts might occur at the early stages of hepatic fat accumulation (Grade 1) and may not necessarily progress in a linear fashion with advancing ultrasonographic severity (Grades 2–3). However, it is important to approach this subgroup result with caution due to the unequal distribution of participants between the disease grades. These outcomes support the notion that the systemic inflammatory burden and lipotoxicity associated with NAFLD could exert a subclinical “mass effect” on the middle ear and Eustachian tube mucosa.

In wideband acoustic immittance evaluations, the Resonance Frequency represents the point where the mass and stiffness components of the middle ear system cancel each other out [10]. Pathologies that increase the stiffness of the tympano-ossicular system characteristically shift the RF to higher frequencies. Conversely, conditions that increase the mass of the middle ear—such as early-stage congestion or mucosal edema—tend to shift the RF toward lower frequencies and predominantly increase absorbance in the low-frequency spectrum [14]. The reduction in RF and the elevated absorbance at 250–500 Hz observed in our NAFLD cohort appear to be consistent with the acoustic profile of a mass-dominated middle ear system. Given our exclusion criteria, which minimized confounding otologic factors and other major systemic inflammatory diseases, it is plausible to consider that this mass effect might be related to subclinical micro-edema within the middle ear mucosa, potentially driven by NAFLD pathophysiology.

The underlying mechanism linking a hepatic metabolic disorder to potential middle ear mucosal alterations might be explored through the lens of lipotoxicity and chronic, low-grade systemic inflammation. NAFLD is increasingly recognized as a systemic condition that can trigger vascular and endothelial dysfunction [15]. In this context, the sphingomyelinase–ceramide pathway could play a contributory role. As extensively described in the recent literature, excessive accumulation of hepatic lipids activates the sphingomyelinase pathway, leading to the systemic circulation of toxic ceramides [16]. Elevated circulating ceramides have been reported to directly disrupt endothelial tight junctions, increase microvascular permeability, and promote the release of pro-inflammatory cytokines [17]. Since the middle ear cavity and the Eustachian tube are lined with a vascularized respiratory mucosa, they are likely susceptible to such circulating inflammatory mediators. One plausible mechanistic explanation for our findings could involve the sphingomyelinase–ceramide pathway, which has been implicated in the systemic lipotoxicity and endothelial dysfunction associated with NAFLD. However, it is important to emphasize that this remains a speculative hypothesis at this stage, as we did not directly measure circulating ceramide levels or serum inflammatory markers (such as CRP), or perform histological analysis of the middle ear mucosa to confirm micro-edema. Future studies incorporating these biomarkers are required to validate this proposed mechanism.

Moreover, our subgroup analysis comparing early-stage (Grade 1) and advanced-stage (Grades 2–3) hepatosteatosis revealed similar biomechanical alterations in both subgroups, suggesting that the mucosal response may be triggered during the initial phases of lipotoxicity rather than exhibiting a dose-dependent progression. Several factors may explain this lack of linearity. First, ultrasonographic grading primarily reflects hepatic fat infiltration and does not reliably distinguish between simple steatosis and steatohepatitis (NASH), as it does not assess inflammation [18]. Since our study used USG-based grading rather than histopathological assessment or inflammatory biomarkers, patients with higher USG grades may have had varying degrees of inflammation. Second, the small sample size in the advanced-stage subgroup (*n* = 13) limits statistical power to detect subtle differences. Third, the cross-sectional design captures a single time point; longitudinal studies are needed to evaluate true progression. Therefore, while our findings suggest an early, non-linear mucosal response, they should be interpreted with caution and validated in larger, histologically or biomarker-stratified cohorts.

From a practical clinical perspective, our findings introduce a novel paradigm for the early screening and interdisciplinary management of NAFLD patients. Traditionally, otologic evaluations are not routine in the clinical pathway of patients with metabolic fatty liver diseases. However, our objective WBT results demonstrate that subclinical middle ear biomechanical alterations occur even in the earliest stages (Grade 1) of hepatic fat accumulation, long before noticeable hearing loss develops. Clinically, this positions wideband tympanometry as a highly sensitive, rapid, and non-invasive screening tool that can detect early metabolic microvascular and mucosal changes in the auditory system. Identifying these subclinical “mass effects” allows clinicians to recognize early otologic risks, implement closer audiological monitoring, and counsel patients on stricter lifestyle modifications to mitigate systemic lipotoxicity before permanent sensory or conductive damage ensues. Furthermore, these results emphasize the need for a collaborative, interdisciplinary approach between gastroenterologists and otolaryngologists for a more comprehensive assessment of metabolic syndrome manifestations.

### Limitations of the Study

While the present study provides preliminary insights into the potential subclinical otologic manifestations of NAFLD, several limitations should be acknowledged. First, the relatively small overall sample size may limit the generalizability of the findings to the broader NAFLD population. More specifically, regarding our subgroup analysis, the limited number of patients in the Grade 1 (*n* = 15) and Grade 2–3 (*n* = 13) categories inherently reduces the statistical power of this comparison. Furthermore, the grouping and potential clinical overlap between Grade 2 and Grade 3 hepatosteatosis led to an unequal distribution of participants among severity grades, which serves as a structural limitation in analyzing advanced disease stages. Consequently, the lack of a statistically significant dose-dependent deterioration should be interpreted with caution, as a larger cohort might be required to detect subtle, progressive biomechanical differences between advancing disease stages. Therefore, larger, multicenter cohorts would be beneficial to further validate these initial observations. Second, the diagnosis and grading of hepatosteatosis were based primarily on abdominal ultrasonography (USG). Although USG is widely used as a first-line imaging modality, it is important to acknowledge its inherent limitations: conventional gray-scale USG cannot reliably differentiate between simple steatosis and steatohepatitis as it does not directly assess the key histologic features of non-alcoholic steatohepatitis (NASH), namely lobular inflammation and hepatocyte ballooning. Therefore, our study reflects an association with the degree of hepatic fat accumulation rather than with the severity of necroinflammation or fibrosis. Third, the healthy control group was significantly younger than the NAFLD cohort. Given that chronological aging can independently influence middle ear compliance, tissue elasticity, and acoustic immittance parameters, this age discrepancy stands as a potential confounding factor. Although we adjusted for age as a covariate in our statistical models to isolate the independent impact of hepatic steatosis, future age-matched cohorts are necessary to completely eliminate the confounding effects of age on wideband tympanometry outcomes. Finally, while we have proposed a pathophysiological mechanism involving systemic lipotoxicity and potential mucosal micro-edema, we did not directly measure circulating pro-inflammatory cytokines, serum ceramide levels, or histological changes in the middle ear mucosa. Therefore, our mechanistic interpretation remains speculative and requires validation in future molecular and histological studies.

## 5. Conclusions

In conclusion, the findings of this study suggest that non-alcoholic fatty liver disease (NAFLD) may be associated with subclinical biomechanical alterations in the middle ear, characterized by a decrease in Resonance Frequency and elevated low-frequency absorbance. These wideband tympanometry patterns point towards a mass-dominated middle ear system, which could potentially be related to mucosal micro-edema driven by the systemic low-grade inflammation inherent to NAFLD. Notably, these biomechanical shifts were observed even in the early stages of hepatic fat accumulation (Grade 1), suggesting that the middle ear mucosa might react to initial metabolic stress. Wideband tympanometry appears to be a valuable, non-invasive tool that could aid in detecting early subclinical otologic changes in patients with systemic metabolic disorders. Ultimately, our findings contribute to the growing body of literature regarding the extrahepatic manifestations of NAFLD, highlighting the potential vulnerability of the middle ear to systemic inflammatory burdens and suggesting a possible role for otologic evaluation in this patient population.

## Figures and Tables

**Table 1:** Demographic and Clinical Characteristics of the Study and Control Groups.

Characteristics	Study Group (*n* = 28)	Control Group (*n* = 30)	*p*-Value
**Age (years)**, Mean ± SD	36.8 ± 7.3	30.7 ± 7.3	0.002 *
**Sex, *n* (%)**			0.746
- Male	18 (64.3%)	17 (56.7%)	
- Female	10 (35.7%)	13 (43.3%)	
**Pure-tone Average (dB HL)**			
- Right Ear (Mean ± SD)	12.6 ± 3.4	11.8 ± 3.1	0.354
- Left Ear (Mean ± SD)	13.1 ± 3.8	12.2 ± 3.5	0.358
**Hepatosteatosis Grade, *n* (%)**			-
- Grade 1	15 (53.6%)	-	
- Grade 2	9 (32.1%)	-	
- Grade 3	4 (14.3%)	-	

* Notes: Pure-Tone Average (calculated across 500, 1000, 2000, and 4000 Hz), dB HL: Decibel Hearing Level SD = Standard Deviation. Statistically significant difference (*p* < 0.05). Age was analyzed using the Independent Samples *t*-test; Sex was analyzed using the Chi-Square test.

**Table 2:** Wideband Tympanometry (WBT) Parameters of the Right Ear.

Parameter	Study Group (*n* = 28)	Control Group (*n* = 30)	*p*-Value
Resonance Frequency (Hz)	781.6 ± 218.7	1010.6 ± 529.0	0.035 *
Middle Ear Pressure (daPa)	−16.0 ± 30.7	−12.8 ± 35.8	0.719
Absorbance at 250 Hz (%)	24.0 ± 8.9	10.3 ± 6.3	<0.001 *
Absorbance at 500 Hz (%)	45.5 ± 15.3	27.1 ± 13.5	<0.001 *
Absorbance at 1000 Hz (%)	74.5 ± 13.4	64.4 ± 18.7	0.021 *
Absorbance at 2000 Hz (%)	73.0 ± 19.1	70.4 ± 15.3	0.572
Absorbance at 4000 Hz (%)	41.4 ± 25.7	53.0 ± 19.6	0.062
Absorbance at 8000 Hz (%)	27.1 ± 15.8	31.4 ± 13.3	0.269
Peak Absorbance (%)	86.4 ± 9.9	84.8 ± 11.0	0.562
Peak Absorbance Frequency (Hz)	1808.8 ± 925.5	2675.3 ± 1501.2	0.010 *

Notes: Values are presented as Mean ± Standard Deviation (SD). Statistical comparisons between the groups were performed using the Independent Samples *t*-test. Statistically significant differences (*p* < 0.05) are indicated with an asterisk (*). Abbreviations: Hz: Hertz; daPa: Decapascal; %: Percentage; *n*: Number of participants.

**Table 3:** Wideband Tympanometry (WBT) Parameters of the Left Ear.

Parameter	Study Group (*n* = 28)	Control Group (*n* = 30)	*p*-Value
Resonance Frequency (Hz)	740.3 ± 212.7	1221.6 ± 754.2	0.002 *
Middle Ear Pressure (daPa)	−17.1 ± 57.8	−10.1 ± 14.6	0.539
Absorbance at 250 Hz (%)	25.4 ± 12.0	10.1 ± 7.8	<0.001 *
Absorbance at 500 Hz (%)	45.3 ± 17.9	25.7 ± 15.6	<0.001 *
Absorbance at 1000 Hz (%)	72.6 ± 16.5	62.4 ± 16.2	0.020 *
Absorbance at 2000 Hz (%)	74.1 ± 13.0	73.1 ± 17.2	0.788
Absorbance at 4000 Hz (%)	46.6 ± 22.7	52.1 ± 22.1	0.360
Absorbance at 8000 Hz (%)	31.7 ± 20.2	32.8 ± 17.3	0.827
Peak Absorbance (%)	86.9 ± 9.2	84.4 ± 13.4	0.402
Peak Absorbance Frequency (Hz)	1975.1 ± 1134.4	2720.5 ± 1219.0	0.019 *

Notes: Values are presented as Mean ± Standard Deviation (SD). Statistical comparisons between the groups were performed using the Independent Samples *t*-test. Statistically significant differences (*p* < 0.05) are indicated with an asterisk (*). Abbreviations: Hz: Hertz; daPa: Decapascal; %: Percentage; *n*: Number of participants.

**Table 4:** Comparison of Key WBT Parameters Between Hepatosteatosis Subgroups.

Parameter	Grade 1 NAFLD (*n* = 15)	Grade 2–3 NAFLD (*n* = 13)	*p*-Value
Right Ear Resonance Frequency (Hz)	767.3 ± 231.1	798.2 ± 211.6	0.715
Left Ear Resonance Frequency (Hz)	749.6 ± 245.0	729.5 ± 177.6	0.804
Right Ear Absorbance at 250 Hz (%)	24.3 ± 6.9	23.7 ± 11.0	0.873
Left Ear Absorbance at 250 Hz (%)	24.8 ± 9.9	26.0 ± 14.4	0.803
Right Ear Absorbance at 500 Hz (%)	49.2 ± 15.0	41.2 ± 15.1	0.174
Left Ear Absorbance at 500 Hz (%)	46.0 ± 17.8	44.5 ± 18.8	0.827
Right Ear Peak Absorbance Freq. (Hz)	1535.5 ± 679.5	2124.1 ± 1089.7	0.108
Left Ear Peak Absorbance Freq. (Hz)	1830.7 ± 807.2	2141.6 ± 1441.8	0.499

Values are presented as Mean ± Standard Deviation. Independent samples *t*-test revealed no statistically significant differences between the subgroups (*p* > 0.05).

## Data Availability

The data presented in this study are available on request from the corresponding author. The data are not publicly available due to ethical and privacy restrictions.
